# Establishing simulation-derived acetabular targets for walking and deep squat: a musculoskeletal modeling study

**DOI:** 10.3389/fbioe.2026.1962946

**Published:** 2026-09-24

**Authors:** Haijun Xu, Zheng Jin, Wei Shi, Aowei Wang, Wenjun Cheng, Zhenhua Zhu

**Affiliations:** 1 Hubei Provincial Sports Medicine Center, Hubei Provincial Clinical Research Center for Orthopaedics, Hubei Key Laboratory of Sports Injury and Precision Therapy, Wuhan Fourth Hospital, Wuhan, China; 2 Department of Clinical Laboratory, Wuhan Fourth Hospital, Wuhan, China

**Keywords:** acetabular component positioning, deep squat, dual-activity feasible zone, musculoskeletal modeling, total hip arthroplasty

## Abstract

**Background:**

Deep squats are required for basic activities of daily life, such as toileting and floor work, but the Lewinnek acetabular safe zone was based on static supine radiographs and did not account for dynamic pelvic positioning during functional activities. Consequently, quantitative positioning targets that integrate walking with deep-squat biomechanics remain poorly defined.

**Methods:**

Simulations were performed in the AnyBody Modeling System using a generic adult musculoskeletal model. Angular Impingement Distance and Angular Edge-loading Distance (AED) were computed at 1° increments across 10,201 acetabular component positions during walking and deep squat. A dual-activity feasible zone was defined as component positions simultaneously satisfying AID- and AED-based criteria for both activities. Sensitivity to femoral head diameter (28, 32, and 36 mm) was evaluated.

**Results:**

In the normal spinal-mobility scenario, the dual-activity feasible zone contained 231 positions, with a target position at radiographic anteversion (RA) 24° and radiographic inclination (RI) 46°. Only 21.6% of these feasible positions fell within the Lewinnek zone; most required higher anteversion or inclination than the Lewinnek target. In the reduced lumbopelvic-mobility scenario, the feasible zone contained 209 positions with a target position shifted toward higher anteversion and inclination (RA 36°, RI 56°) and no overlap with the Lewinnek zone. Sensitivity to femoral head diameter was substantial: compared with the 28-mm head, the 36-mm head increased the feasible zone 4.2-fold (375 vs. 89 positions), with the 32-mm default yielding 231 positions.

**Conclusion:**

We present a computational framework for deriving activity-specific acetabular positioning targets from dynamic musculoskeletal simulations of walking and deep squat. The resulting targets are biomechanically grounded but remain simulation-derived hypotheses pending clinical validation.

## Introduction

Total hip arthroplasty (THA) is one of the most successful surgical procedures in modern orthopaedic medicine (Learmonth et al., 2007). As the procedure has matured, patient expectations have evolved beyond pain relief and basic ambulation toward the resumption of demanding functional activities, including deep squatting for toileting, floor seating, and occupational activities (Nawatthakul et al., 2022).

The Lewinnek acetabular safe zone (radiographic inclination [RI] 40° ± 10°, radiographic anteversion [RA] 15° ± 10°) was derived from static supine radiographs (Lewinnek et al., 1978). Abdel et al. (Abdel et al., 2016) reported that 58% of dislocated THAs fell within this zone, casting doubt on whether a single static window can reliably predict stability across diverse patients and activities. The fundamental issue is that supine radiographic orientation fails to capture dynamic pelvic positioning during functional movement. During deep squat, for example, pelvic posterior tilt increases substantially relative to standing or sitting (Lazennec et al., 2015), but the Lewinnek zone does not account for the dynamic pelvic orientations and extreme positions these activities require. This mismatch between static radiographic targets and dynamic functional demands may explain why a subset of conventionally placed cups were associated with impingement or instability during deep squat activities (Lazennec et al., 2015; Tezuka et al., 2019).

Recent studies have shifted toward personalized acetabular positioning algorithms that account for individual anatomy and functional demands. One line of development has focused on functional safe zones. These incorporate dynamic spinopelvic biomechanics during standing and sitting (Heckmann and Lieberman, 2021; Koutalos et al., 2024), as formalized through concepts such as the combined sagittal index (Grammatopoulos et al., 2022). A second line has focused on anatomy-driven planning tools. Combined anteversion rules (Wan et al., 2009) and no-impingement algorithms were used to tailor cup orientation to patient-specific skeletal geometry (Tang et al., 2022; Jin et al., 2025). Despite these advances, most current frameworks remain constrained. They rely on static or quasi-static poses rather than continuous motion data from real patient activities (Grammatopoulos et al., 2022), and they focus primarily on avoiding impingement while giving limited attention to edge-loading (Danaei and McPhee, 2022; Miki et al., 2014). This is an important limitation because lower-limb muscle forces can direct the hip joint reaction force toward the acetabular rim even in the absence of neck-rim contact (Etchels et al., 2025), with consequences for both instability and bearing wear (Mellon et al., 2013). A method that evaluates both impingement and edge-loading throughout dynamic activities would address these gaps.

The present study applies a musculoskeletal modeling framework that evaluates acetabular component positioning through two complementary metrics. The Angular Impingement Distance (AID) quantifies the angular margin to implant impingement between the femoral neck and the acetabular liner. The Angular Edge-loading Distance (AED) quantifies the angular margin by which the hip joint reaction force approaches the acetabular rim. Using a validated musculoskeletal model, we simulated continuous walking and deep-squat trajectories and computed AID and AED across a broad grid of acetabular component positions. We compared a normal spinal-mobility scenario with a reduced lumbopelvic-mobility scenario, and we evaluated the sensitivity of the resulting feasible zones to femoral head diameter. By integrating impingement and edge-loading assessment across dynamic activities, this work establishes a simulation-based method for deriving anatomy- and activity-specific positioning targets.

## Materials and methods

All simulations were conducted in the AnyBody Modeling System (version 8.0.4; AnyBody Technology A/S, Aalborg, Denmark) using the TLEM 2.0 (Twente Lower Extremity Model) full-body musculoskeletal model as distributed in the AnyBody Managed Model Repository (AMMR) version 3.0.4 (Carbone et al., 2015). The TLEM 2.0 dataset was derived from medical imaging and cadaver dissection, providing anatomically detailed lower-extremity geometry for musculoskeletal modeling (Carbone et al., 2015). The AnyBody/TLEM 2.0 framework has previously been validated against *in vivo* hip contact force measurements from instrumented prostheses and showed good consistency between predicted and measured forces (De Pieri et al., 2018). It has also been used to quantify hip and knee joint kinetics during demanding activities such as deep squat and forward lunge (De Roeck et al., 2020). The model represents a 70 kg, 175 cm adult male with 162 Hill-type musculotendinous units and 300 mechanical degrees of freedom. A right total hip arthroplasty was implemented in the model. Standard femoral geometric parameters were: neck-shaft angle 130°, femoral anteversion 15°, femoral valgus angle 5°, femoral neck diameter 12 mm, and default femoral head diameter 32 mm. This is an *in silico* computational study; no human participants, patient data, or biological samples were involved, and therefore institutional review board approval was not required.

### Activities and data collection

Two activities were simulated. Walking was modeled at 1.2 m/s using the AnyMoCap Plug-in-Gait framework (AnyBody Technology A/S), a standard reference gait model. The simulation comprised complete gait cycles (total duration 1.83 s, time step 0.01 s, estimated cadence 98 steps/min). Hip flexion ranged from 18° of extension (−18°) to 38° flexion (ROM 56°), hip abduction from 12° adduction to 6° abduction, and pelvis sagittal tilt (PelvisRotZ) from 10° to 17° (ROM 7°). Peak joint reaction force was 2,912 N (4.2 times body weight), with cyclic loading during the stance phase ranging from 0.3 to 4.2 times body weight. Deep squat was simulated over a 3.0-s cycle (time step 0.033 s) with knee flexion driven by a Fourier cosine driver from 10° to 120° (mean 65°, amplitude 55°) (Figure 1a). The feet were positioned at 0.8 times shoulder width with 0° external rotation. Hip flexion reached a maximum of 111° (ROM 91°). Pelvis sagittal tilt (PelvisRotZ) ranged from −16° to −5° (ROM 10.5°). For both activities, lumbar motion was coupled to hip flexion via PelvisThoraxExtension = −Coef × HipFlexion, where Coef = 0.4 in the normal spinal-mobility scenario and Coef = 0.15 in the reduced-lumbopelvic-mobility scenario. In the walking model, the same coupling law was imposed through the lumbopelvic driver of the gait simulation, so that reduced mobility increased the hip-flexion demand and attenuated pelvic sagittal motion during gait (Table 1). AnyBody-based musculoskeletal modeling has been validated for computing lumbar spine loads and lumbopelvic kinematics (Bassani et al., 2017). Reducing this coefficient attenuates the lumbar contribution to hip flexion, forcing the pelvis to compensate through increased sagittal-plane rotation. Peak joint reaction force was 2,261 N (3.3 times body weight).

**FIGURE 1:**
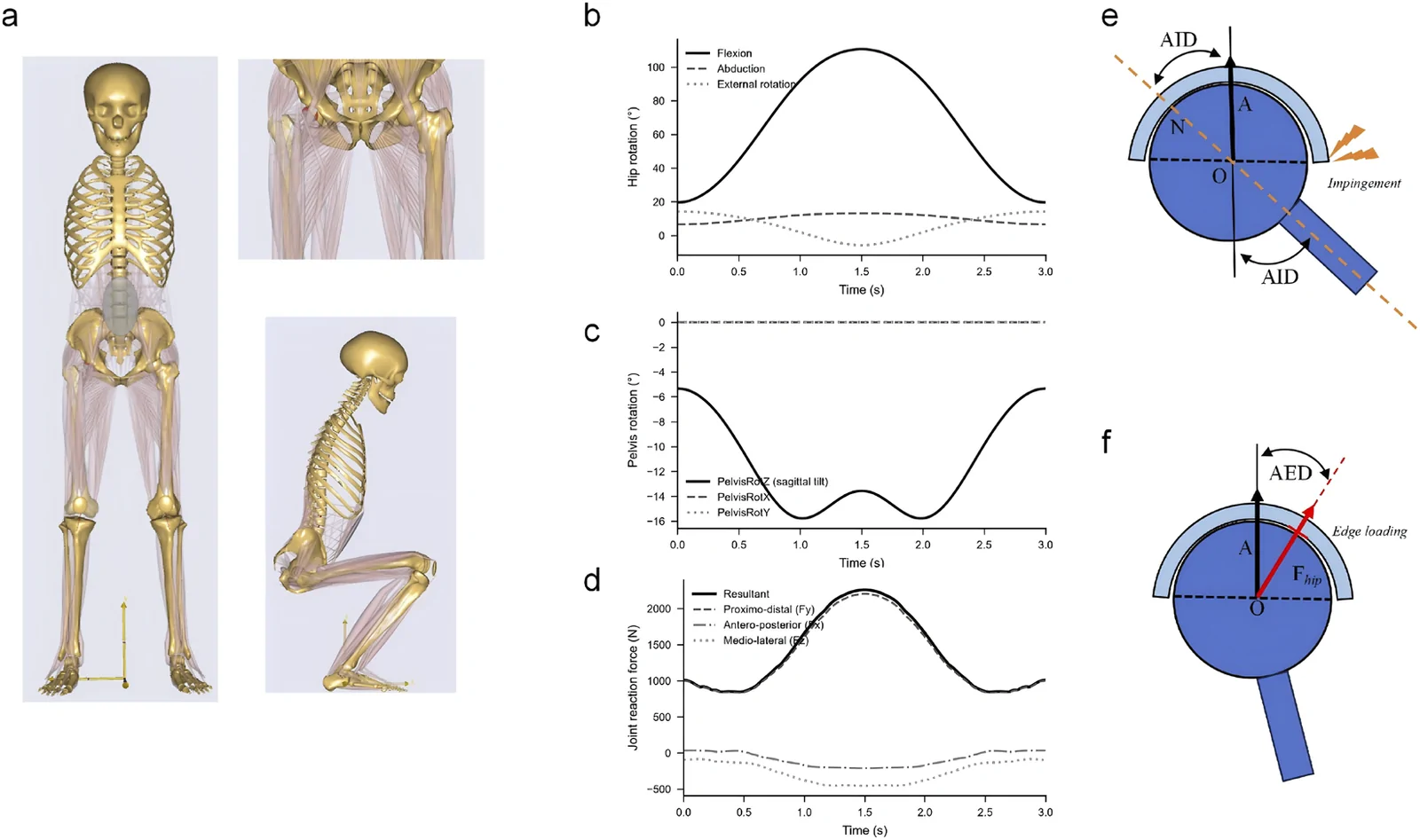
Study overview and simulation framework. **(a)** Musculoskeletal modeling of the deep-squat task in the AnyBody Modeling System using the TLEM 2.0 full-body model with a right total hip arthroplasty. **(b–d)** Simulated time-series outputs during one deep-squat cycle in the normal spinal-mobility scenario: **(b)** hip joint rotation (flexion, abduction, and external rotation), **(c)** pelvic rotation (PelvisRotX, PelvisRotY, and PelvisRotZ, with PelvisRotZ representing sagittal-plane tilt), and **(d)** hip joint reaction force (resultant and three orthogonal components). **(e)** Angular Impingement Distance (AID) algorithm: AID was defined as the maximum angular deviation between the femoral neck axis (oN) and the acetabular opening axis (OA) across all time points; component positions with AID ≤ AIDlimit were considered free of neck–liner impingement. **(f)** Angular Edge-loading Distance (AED) algorithm: AED was defined as the maximum angular deviation between the normalized hip joint reaction force vector and the OA across all time points; positions with AED ≤ 65° were considered free of edge-loading.

**TABLE 1:** Peak kinematic and kinetic values during walking and squat activities.

Parameter	Normal spinal-mobility (Walking)	Normal spinal-mobility (Squat)	Reduced lumbopelvic-mobility (Walking)	Reduced lumbopelvic-mobility (Squat)
Peak hip flexion (°)	37.7	110.7	54.3	121.6
PelvisRotZ ROM (°)	7.0	10.5	5.7	19.9
Peak joint force (N)	2,912	2,261	2,613	1995
Force (× BW)	4.2	3.3	3.8	2.9

Abbreviations: ROM = range of motion; PelvisRotZ = pelvic sagittal-plane rotation (AnyBody coordinate system); BW = body weight (70 kg × 9.81 m/s^2^ = 686.7 N). Peak joint force represents the resultant magnitude of the hip joint reaction force vector. Force values exceeding 200 N were included in the walking analysis to exclude low-force swing-phase intervals; all time points were included in the squat analysis because squat is a continuously loaded activity.

For each activity, time-series data were extracted from the AnyBody output at every simulation time point. Hip joint reaction forces were obtained in three orthogonal components: anterior-posterior (Fx), proximo-distal (Fy), and medio-lateral (Fz). Hip joint angles (flexion, abduction, external rotation) were obtained from the ISB-recommended joint coordinate system. Pelvic orientation angles (RotX, RotY, RotZ) were extracted to define the time-varying transformation between the pelvic anatomical frame and the global reference frame (Figures 1b–d).

### Coordinate transformations and AID and AED computation

A right-handed global coordinate system was adopted, with the X-axis pointing anteriorly, Y-axis proximally, and Z-axis to the right. All subsequent transformations operate within this unified reference frame. Pelvic and femoral anatomical coordinate systems followed the ISB standard (Wu et al., 2002). The acetabular opening axis (OA) was defined in a pelvic-fixed anatomical frame parameterized by radiographic anteversion (RA) and radiographic inclination (RI) (Equation 1):

$${\vec{OA}}_{\text{local}}=\left[\begin{array}{l} \sin\left(\text{RA}\right)\\ -\cos\left(\text{RI}\right)\cos\left(\text{RA}\right)\\ \sin\left(\text{RI}\right)\cos\left(\text{RA}\right) \end{array}\right]$$ (1)

This local vector was transformed to global coordinates at each time point through the pelvic rotation matrix using the Z-Y-X Euler angle sequence (RotZ → RotY → RotX) following the ISB standard (Equation 2). Because the acetabular opening axis is parameterized in the pelvic-fixed anatomical frame, its global orientation follows the pelvis rigidly, whereas hip joint angles are computed independently as femur-relative-to-pelvis rotations in the ISB joint coordinate system; pelvic motion is therefore applied exactly once, through the pelvic rotation matrix acting on the cup-fixed axis, and is not double counted in the relative hip kinematics.

$$\vec{OA}\left(t\right)=R_{z}\left({\text{PelvisRotZ}}_{t}\right)\cdot R_{y}\left({\text{PelvisRotY}}_{t}\right)\cdot R_{x}\left({\text{PelvisRotX}}_{t}\right)\cdot {\vec{OA}}_{\text{local}}$$ (2)

The femoral neck axis (oN) was defined in a femur-fixed frame from implant geometry (neck-shaft angle NSA = 130°, femoral anteversion FAA = 15°, femoral valgus angle = 5°) (Equations 3–5):

$$\theta =180^{{}^{\circ}}-\text{NSA}-\text{abd}\_\text{angle}$$ (3)

$$k=\frac{1}{\sqrt{1+\sin^{2}\theta \cdot \tan^{2}\left(\text{FAA}\right)}}$$ (4)

$${\vec{oN}}_{\text{local}}=\left[\begin{array}{l} -k\sin\theta \tan\left(\text{FAA}\right)\\ -k\cos\theta \\ k\sin\theta \end{array}\right]$$ (5)

It was transformed to global coordinates via the hip joint rotation matrix following the ISB Z-X-Y sequence. AnyBody exports hip external rotation as positive, whereas the ISB convention defines internal rotation as positive; therefore, a negated sign was applied (Equation 6).

$$\vec{oN}\left(t\right)=R_{z}\left({\text{Flexion}}_{t}\right)\cdot R_{x}\left({\text{Abduction}}_{t}\right)\cdot R_{y}\left(-{\text{ExtRot}}_{t}\right)\cdot {\vec{oN}}_{\text{local}}$$ (6)

The hip joint reaction force vector (Fx, Fy, Fz) was obtained from inverse-dynamics analysis and normalized to unit direction (Equation 7):

$$\hat{\vec{F}}\left(t\right)=\frac{\left[F_{x}\left(t\right),F_{y}\left(t\right),F_{z}\left(t\right)\right]}{\sqrt{F_{x}^{2}\left(t\right)+F_{y}^{2}\left(t\right)+F_{z}^{2}\left(t\right)}}$$ (7)

AID was computed as the maximum angular deviation between the femoral neck axis and the acetabular opening axis across all time points (Equation 8) (Figure 1e):

$$\text{AID}=\max_{t}\left[\arccos\left(\vec{oN}\left(t\right)\cdot \vec{OA}\left(t\right)\right)\right]\times \frac{180}{\pi }$$ (8)

AED was computed as the maximum angular deviation between the normalized hip joint reaction force vector and the acetabular opening axis across all time points (Equation 9) (Figure 1f):

$$\text{AED}=\max_{t}\left[\arccos\left(\hat{\vec{F}}\left(t\right)\cdot \vec{OA}\left(t\right)\right)\right]\times \frac{180}{\pi }$$ (9)

For walking, these metrics were calculated at time points where force magnitude exceeded 200 N (selected to exclude low-force swing-phase intervals where edge-loading is not clinically relevant); for deep squat, all time points were included. The extrema underlying these metrics were temporally broad: within ±2 time frames (±0.067 s) of each squat peak, hip flexion, pelvic sagittal tilt, and joint reaction force deviated by no more than 0.3%, 1.1%, and 0.5% of their peak values, respectively, indicating that the maximum-based metrics are insensitive to isolated-frame artifacts.

The maximum angular gap permitted between the femoral neck axis (oN) and the acetabular opening axis (OA) to avoid neck–acetabular rim contact depends on the neck-to-head diameter ratio (Equation 10):

$${\text{AID}}_{\text{limit}}=90^{{}^{\circ}}-\arcsin\left(\frac{d_{\text{neck}}}{D_{\text{head}}}\right)\times \frac{180}{\pi }$$ (10)

### Dual-activity feasible zone definition and grid search

The safety threshold was AID ≤ AID_limit_ and AED ≤65° for both activities. The AID and AED concepts were adopted from the framework proposed by Danaei and McPhee (Danaei and McPhee, 2022), which defines angular impingement distance and angular edge-loading distance to quantify the risk of implant impingement and edge-loading during dynamic activities. An AED threshold of 65° was selected for this study to define a conservative boundary between edge-loading-prone and edge-loading-safe positions. A 1° grid search (10,201 positions) was carried out for RA from −20° to 80° and RI from −10° to 90°. The dual-activity feasible zone was defined as the intersection of the walking and squat feasible zones: component positions that simultaneously satisfied the AID- and AED-based criteria for both activities. The target position was defined as the geometric center (mean RA, mean RI) of all positions within the dual-activity feasible zone. As a robustness check, we also computed the maximum margin Chebyshev center for each zone, defined as the feasible point that maximizes the minimum Euclidean distance to any infeasible point in the RA and RI plane. Sensitivity to femoral head diameter (28 mm, 32 mm, 36 mm) was evaluated. Data aggregation and grid evaluation were performed in Python 3.12.0 (NumPy, pandas, Matplotlib); because the analysis was a deterministic grid evaluation, no inferential statistical tests were performed.

## Results

### Kinematic and kinetic characteristics

Walking kinematics in the normal spinal-mobility scenario showed peak hip flexion of 37.7° and a PelvisRotZ range of motion (pelvic sagittal-plane rotation) of 7.0°. In the reduced-lumbopelvic-mobility scenario, peak hip flexion increased to 54.3° and PelvisRotZ range of motion decreased to 5.7°. During deep squat, peak hip flexion reached 110.7° with a PelvisRotZ range of motion of 10.5° in the normal spinal-mobility scenario, compared with 121.6° and 19.9° in the reduced-lumbopelvic-mobility scenario. Peak joint reaction forces were 2,912 N (4.2 × body weight) during walking and 2,261 N (3.3 × body weight) during deep squat in the normal spinal-mobility scenario, and 2,613 N (3.8 × body weight) and 1,995 N (2.9 × body weight), respectively, in the reduced-lumbopelvic-mobility scenario (Table 1).

### Dual-activity feasible zones

For the normal spinal-mobility scenario, the walking feasible zone contained 3,193 positions and the squat feasible zone contained 2,253 positions. Their intersection, the dual-activity feasible zone, comprised 231 positions (2.26% of the 10,201-position grid), with a target position at radiographic anteversion (RA) 24° and radiographic inclination (RI) 46° (Figure 2). The maximum-margin (Chebyshev) center of this zone (RA 25°, RI 46°; boundary margin 4.2°) lay within 0.7° of the centroid (margin 3.6°), confirming that the centroid is near-optimal with respect to boundary clearance.

**FIGURE 2:**
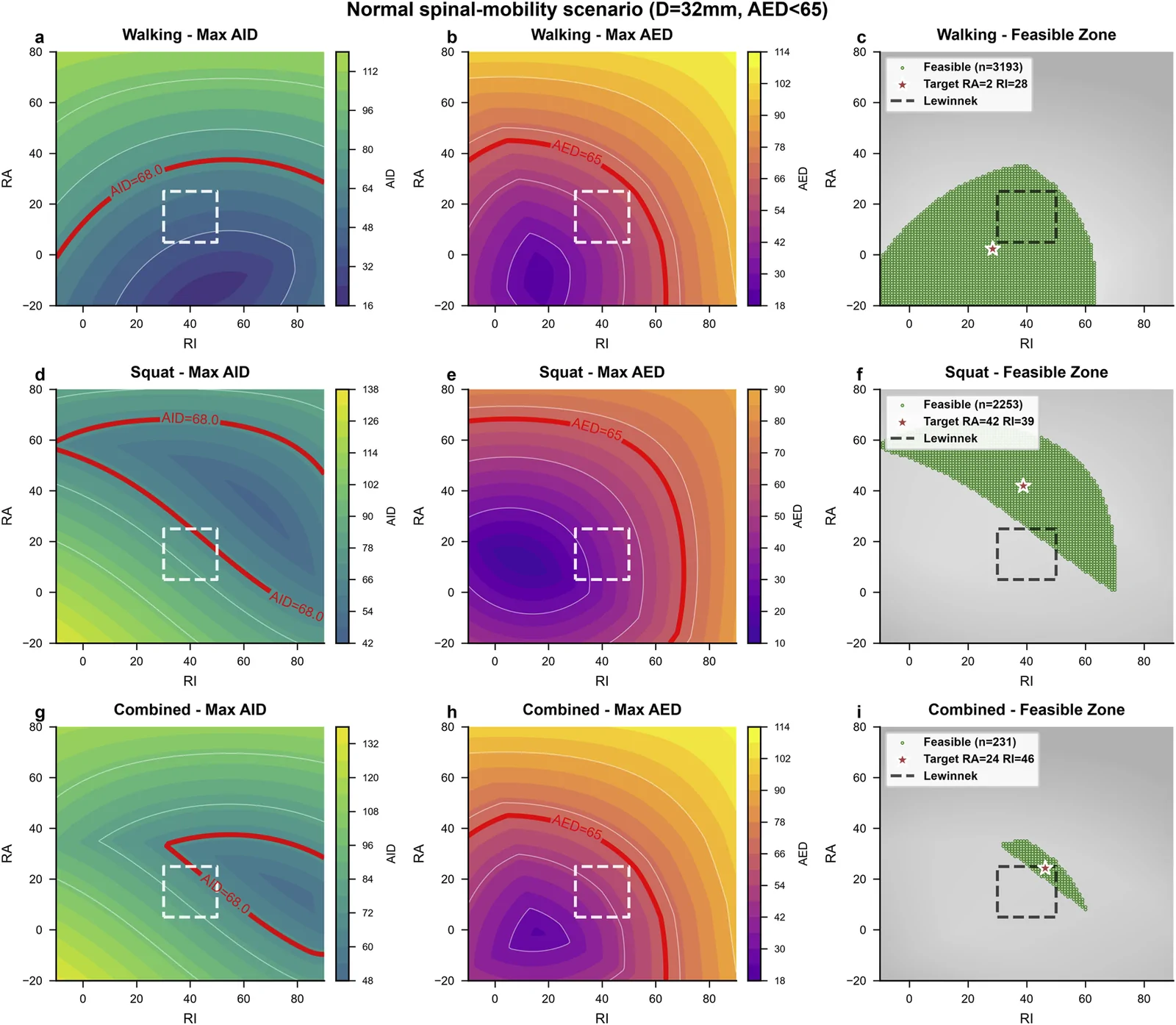
Acetabular component-position analysis in the normal spinal-mobility scenario. Contour maps of maximum AID (left column) and maximum AED (middle column), and the corresponding feasible positions (right column), for walking **(a–c)**, deep squat **(d–f)**, and the dual-activity intersection **(g–i)**. Red contour lines indicate the AID (67.98°, 32-mm head) and AED (65°) thresholds; the dashed rectangle indicates the Lewinnek zone (RA 5°–25°, RI 30°–50°). In the right column, each green dot denotes one component position satisfying both the AID and AED criteria, and the red star marks the target position (geometric center of the feasible zone). The single-activity feasible zones contained 3,193 (walking, **(c)**) and 2,253 (squat, **(f)**) positions; their intersection, the dual-activity feasible zone **(i)**, contained 231 positions with a target at RA 24° and RI 46°; the maximum-margin (Chebyshev) center (RA 25°, RI 46°) coincided with the centroid within one grid step (0.7°). AID, Angular Impingement Distance; AED, Angular Edge-loading Distance; RA, radiographic anteversion; RI, radiographic inclination.

For the reduced-lumbopelvic-mobility scenario, the walking feasible zone contained 5,504 positions and the squat feasible zone contained 589 positions. The dual-activity feasible zone comprised 209 positions (2.05% of the grid), with a target position at RA 36° and RI 56°; its maximum-margin center (RA 35°, RI 58°; margin 3.6°) lay within 1.8° of the centroid (margin 2.2°). The reduction in the squat feasible zone from 2,253 to 589 positions reflects the biomechanical consequence of restricted lumbopelvic mobility (Figure 3).

**FIGURE 3:**
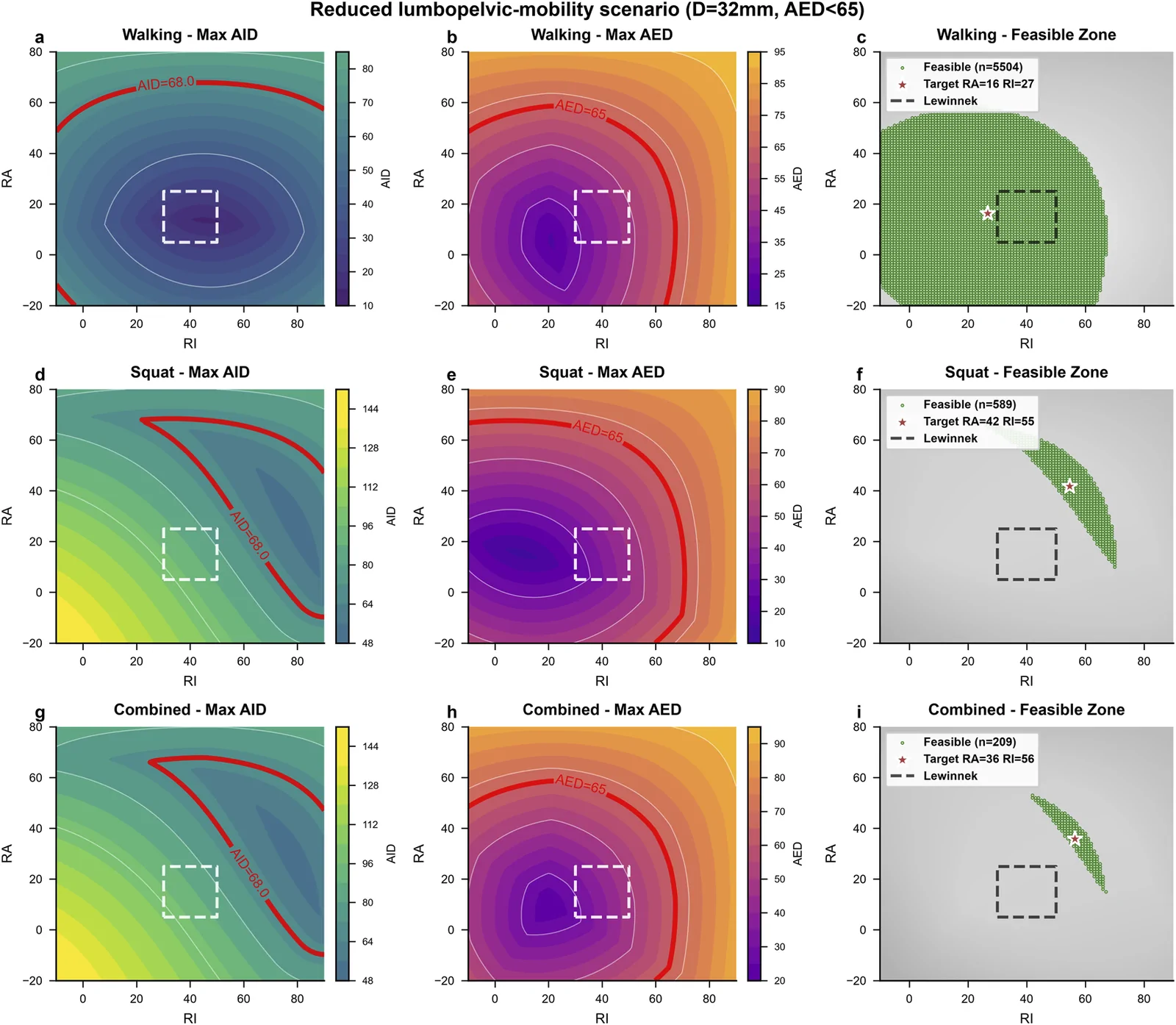
Acetabular component-position analysis in the reduced lumbopelvic-mobility scenario. Panel layout: walking **(a–c)**, deep squat **(d–f)**, and the dual-activity intersection **(g–i)**, with identical thresholds (AID 67.98°, AED 65°) and the Lewinnek zone (dashed rectangle). Reducing the lumbar contribution to hip flexion expanded the walking feasible zone to 5,504 positions **(c)** but markedly reduced the squat feasible zone to 589 positions **(f)**. The resulting dual-activity feasible zone **(i)** contained 209 positions with a target at RA 36° and RI 56° and showed no overlap with the Lewinnek zone; the maximum-margin center (RA 35°, RI 58°) lay within 1.8° of the centroid. AID, Angular Impingement Distance; AED, Angular Edge-loading Distance; RA, radiographic anteversion; RI, radiographic inclination.

### Comparison with the Lewinnek safe zone

In the normal spinal-mobility scenario, 50 of the 231 dual-activity feasible positions (21.6%) fell within the Lewinnek zone (RA 5°–25°, RI 30°–50°). The remaining 181 positions (78.4%) required component orientations outside the Lewinnek zone, predominantly toward higher anteversion, higher inclination, or both. Only 11.3% of all positions within the Lewinnek zone (50 of 441) were dual-activity feasible. Failure mode decomposition showed that every infeasible Lewinnek position failed the AID criterion alone: all 391 in the normal scenario and all 441 in the reduced scenario. None failed the AED criterion alone or both criteria. Across the full grid, infeasible positions in the normal scenario comprised 3,916 AID-only, 1,352 AED-only, and 4,702 dual-criterion failures; the corresponding numbers in the reduced scenario were 5,319, 2,090, and 2,583. A per-activity breakdown is provided in Supplementary Table S1. In the reduced-lumbopelvic-mobility scenario, none of the 209 dual-activity feasible positions overlapped with the Lewinnek zone. The target position (RA 36°, RI 56°) lay outside both the anteversion and inclination maxima of the Lewinnek target.

### Femoral head diameter sensitivity

Femoral head diameter substantially influenced the size of the dual-activity feasible zone in the normal spinal-mobility scenario (Table 2). With a 28-mm head (AID limit 64.62°), the dual-activity zone contained 89 positions. With the default 32-mm head (AID limit 67.98°), the zone expanded to 231 positions. With a 36-mm head (AID limit 70.53°), the zone further increased to 375 positions, representing a 1.62-fold increase relative to the 32-mm head and a 4.2-fold increase relative to the 28-mm head. The target position varied only slightly with head diameter (RA 24°–25°, RI 45°–48°), remaining close to the 32-mm default target (RA 24°, RI 46°) (Figure 4).

**TABLE 2:** Effect of femoral head diameter on dual-activity feasible zone parameters (Normal spinal-mobility scenario).

Femoral head diameter (mm)	AID limit (°)	Dual-activity feasible positions	Target RA (°)	Target RI (°)	Fold vs 32 mm	Max-margin center RA/RI (°)
28	64.62	89	25	48	0.39	26/48
32	67.98	231	24	46	1.00	25/46
36	70.53	375	24	45	1.62	26/43

Abbreviations: AID = angular impingement distance; RA = radiographic anteversion; RI = radiographic inclination. Target position is the geometric center (mean RA, mean RI) of all positions within the dual-activity feasible zone. AID limit = 90° − arcsin (d_neck / D_head) × 180/π, where d_neck = 12 mm. Fold vs 32 mm = ratio of dual-activity feasible positions for the given head diameter relative to the 32-mm head (231 positions). The dual-activity feasible zones comprised 0.87%, 2.26%, and 3.68% of the 10,201-position grid for the 28-, 32-, and 36-mm heads, respectively. The maximum-margin (Chebyshev) centers were RA, 26°/RI, 48°, RA, 25°/RI, 46°, and RA, 26°/RI, 43° for the three head diameters, within 0.6–3.0° of the corresponding centroids.

**FIGURE 4:**
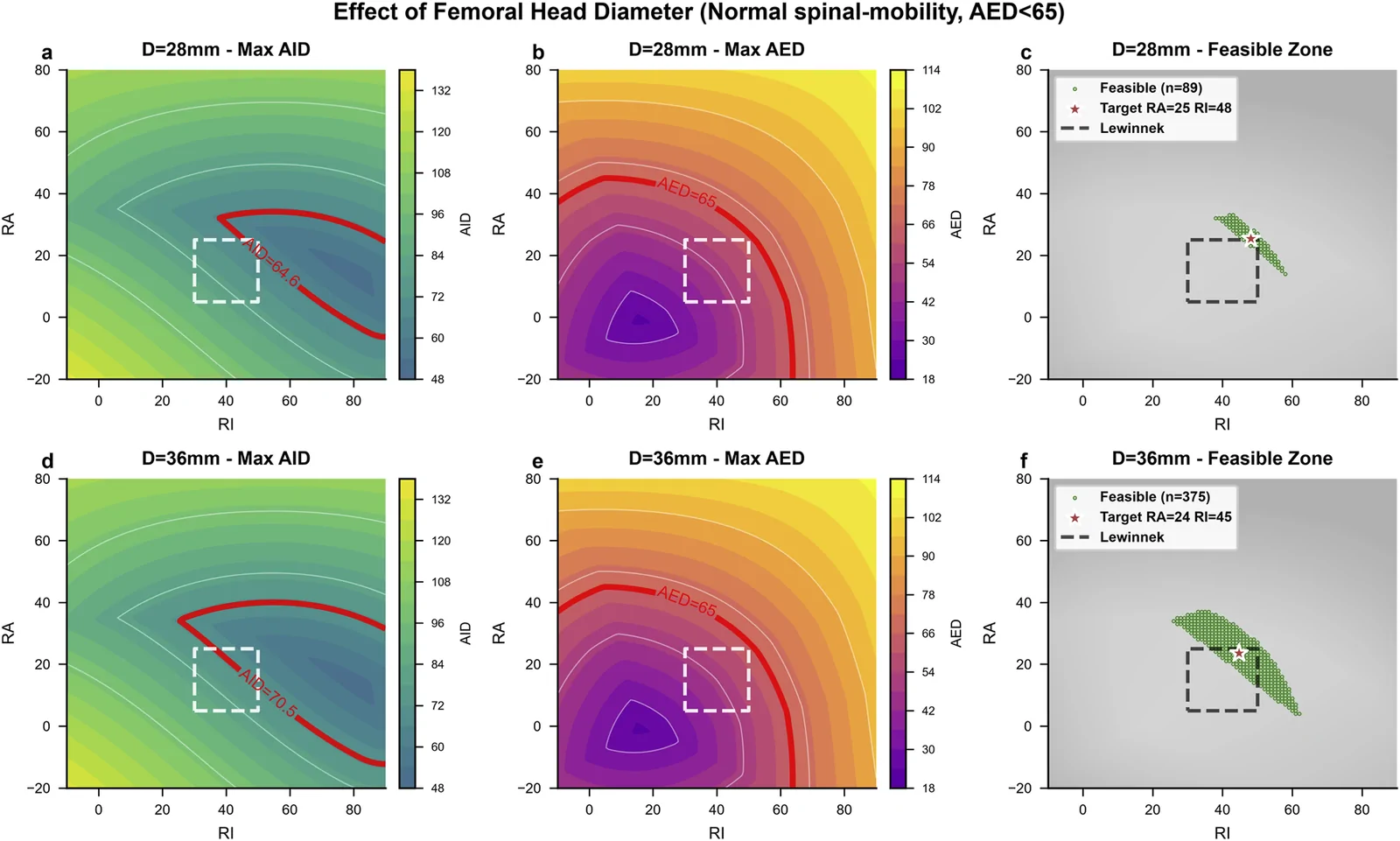
Effect of femoral head diameter on the dual-activity feasible zone in the normal spinal-mobility scenario. Rows correspond to 28-mm **(a–c)** and 36-mm **(d–f)** femoral heads, with diameter-dependent AID limits of 64.62° and 70.53°, respectively (red contour lines); the AED threshold was 65° throughout. Results for the default 32-mm head (AID limit 67.98°) are shown in Figure 2. Left and middle columns show maximum AID and AED for the combined (dual-activity) analysis; the right column shows the dual-activity feasible positions (green dots) and target position (red star), with the dashed rectangle indicating the Lewinnek zone. The feasible zone contained 89 and 375 positions for the 28- and 36-mm heads, respectively (231 positions for the 32-mm default), whereas the target position varied only slightly (RA 24°–25°, RI 45°–48°). AID, Angular Impingement Distance; AED, Angular Edge-loading Distance; RA, radiographic anteversion; RI, radiographic inclination.

## Discussion

Integrating continuous walking and deep-squat biomechanics substantially redefines the acetabular component targets required for dual-activity function after total hip arthroplasty. In the normal spinal-mobility scenario, the dual-activity feasible zone centered at RA 24° and RI 46° overlapped with the Lewinnek zone at only 21.6% of its positions (50 of 231); conversely, only 11.3% of Lewinnek positions were dual-activity feasible. In the reduced-lumbopelvic-mobility scenario, the target shifted to RA 36° and RI 56° and did not intersect the Lewinnek zone at all. These results support the growing consensus that static radiographic safe zones are inadequate predictors of dynamic stability (Abdel et al., 2016).

In the normal spinal-mobility scenario, the target sits near the upper margin of the Lewinnek window. This explains why a subset of conventionally placed cups permit squatting: the Lewinnek window is broad enough that some higher-anteversion, higher-inclination positions happen to satisfy deep-squat biomechanics. However, most Lewinnek positions failed because of impingement risk, not edge-loading. The lower-inclination portion of the Lewinnek target (RI < 40°) could not accommodate the deep hip flexion required for squatting. This pattern aligns with Tezuka et al.'s finding that functional safe zones are superior to the Lewinnek target for predicting stability (Tezuka et al., 2019), and with Lazennec et al.'s observation that functional cup orientation changes markedly between standing, sitting and squatting (Lazennec et al., 2015). By quantifying the mismatch between static radiographic zones and dynamic simulation-derived targets, our study provides a biomechanical rationale for moving beyond one-size-fits-all positioning.

Two design choices of the feasible zone warrant clarification. First, the zone is intended for patients for whom deep-squat capability is functionally non-negotiable, such as those whose toileting and daily activities require squatting; for this population a single squat failure is not offset by thousands of successful gait cycles, so a conservative intersection rather than a frequency-weighted average is the appropriate criterion. Second, treating impingement and edge-loading as equally weighted pass/fail criteria does not materially affect the present zones: within and around the Lewinnek zone, failures were exclusively impingement-type (391 of 441 Lewinnek positions failed the AID criterion alone in the normal scenario, and all 441 in the reduced scenario), so a severity-weighted formulation prioritizing impingement would leave the zones essentially unchanged. Frequency- or severity-weighted continuous risk metrics, which may be more suitable for the general THA population, remain an objective for future work.

Restricted lumbopelvic mobility had the most pronounced effect. When lumbar contribution to hip flexion was reduced, the pelvis had to tilt further posteriorly to accommodate the squat, and the feasible target moved to substantially higher anteversion and inclination. This agrees with clinical evidence that abnormal spinopelvic mobility is a major contributor to THA instability (Heckmann and Lieberman, 2021; Haffer et al., 2020), and suggests that patients with stiff spines may need more aggressive component positioning to achieve deep-squat function. Such positions exceed conventional boundaries, however, and require careful clinical validation before they can be translated.

Femoral head diameter modulated feasible zone size but not its target position. Larger heads increased the AID limit and expanded the zone four-fold, whereas the target position remained close to RA 24° and RI 46° across all head sizes. This is consistent with prior work showing that larger heads reduce prosthetic impingement (Waddell et al., 2019), and indicates that head size modulates the tolerance around the target orientation, rather than shifting the target orientation itself.

Beyond these specific targets, the framework offers a template for patient-specific planning. Preoperative CT could supply individual pelvic and femoral anatomy, and motion capture could record patient-specific gait and squat trajectories; the inverse-dynamics pipeline would then yield a feasible zone shaped by both skeletal geometry and intended activities. The concept of patient-specific functional safe zones is gaining traction, with early clinical data showing reduced dislocation rates when orientation is tailored to individual motion (Sharma et al., 2021). Simulation-based tools could eventually feed into navigation or robotic systems to place components within computed boundaries. This consideration is practically relevant because the maximum boundary margins of the present zones (3.6°–4.2°) are smaller than typical freehand placement error, which reinforces the need for guided implantation if such targets are to be realized clinically. The present implementation is generic, but it establishes the computational proof of principle for activity-specific planning.

This study has several limitations. The analysis used a generic musculoskeletal model and generic squat kinematics, so the reported targets should be interpreted as simulation-derived hypotheses rather than surgical recommendations. We did not model implant-specific geometry, bearing surface, soft-tissue tension or muscle deficits, all of which can shift the effective safe zone. The AED threshold was selected for this study and should be validated against clinical outcomes. Finally, the proposed inclination values exceed conventional ranges; their safety with respect to wear, impingement and dislocation must be confirmed in patient cohorts before adoption.

## Conclusion

Integrating deep squat with walking redefines the acetabular component targets required for dual-activity function after total hip arthroplasty. The dual-activity feasible zone provides simulation-derived targets that may better accommodate the biomechanical demands of both activities than the Lewinnek zone. Reduced lumbopelvic mobility shifts the target position toward higher anteversion and inclination, and femoral head diameter modulates the size of the feasible zone without substantially altering the target position. Although these targets were derived from a generic model, they provide a foundation for developing patient-specific, activity-based planning strategies in total hip arthroplasty.

## Data Availability

The original contributions presented in the study are included in the article/Supplementary Material, further inquiries can be directed to the corresponding authors.
