# Supplementary material for: Establishing simulation-derived acetabular targets for walking and deep squat: a musculoskeletal modeling study
Source: Front Bioeng Biotechnol. 2026 Sep 24;14:1962946. doi: 10.3389/fbioe.2026.1962946 (PMC13646117; doi:10.3389/fbioe.2026.1962946)
Supplement: Supplementary file 1 [file Table1.docx]

**Supplementary Table S1. Failure-mode decomposition of acetabular component positions by activity and mobility scenario.**

Number of grid positions (of 10,201) excluded by the AID criterion alone, the AED criterion alone, or both criteria, and the number satisfying both criteria (feasible). AID limit 67.98° (32-mm head); AED threshold 65°.

| **Scenario / Activity** | **AID-only failure** | **AED-only failure** | **Both criteria failed** | **Feasible (AID+AED)** | **Total** |
| --- | --- | --- | --- | --- | --- |
| Normal spinal-mobility / Walking | 954 | 1,811 | 4,243 | 3,193 | 10,201 |
| Normal spinal-mobility / Squat | 4,309 | 1,744 | 1,895 | 2,253 | 10,201 |
| Normal spinal-mobility / Dual-activity (intersection) | 3,916 | 1,352 | 4,702 | 231 | 10,201 |
| Reduced lumbopelvic-mobility / Walking | 37 | 2,956 | 1,704 | 5,504 | 10,201 |
| Reduced lumbopelvic-mobility / Squat | 5,885 | 1,738 | 1,989 | 589 | 10,201 |

*Within the Lewinnek zone (RA 5–25°, RI 30–50°; 441 positions): normal scenario — 391 AID-only failures, 0 AED-only, 0 both, 50 feasible; reduced scenario — 441 AID-only failures, 0 feasible.*
